# Sociodemographic factors associated with acceptance of COVID-19 vaccine and clinical trials in Uganda: a cross-sectional study in western Uganda

**DOI:** 10.1186/s12889-021-11197-7

**Published:** 2021-06-10

**Authors:** Isaac Echoru, Patricia Decanar Ajambo, Emmanuel Keirania, Edmund E. M. Bukenya

**Affiliations:** 1 School of Medicine, Kabale University, Kabale, Uganda; 2 Faculty of Clinical Medicine and Dentistry, Kampala International University, western Campus, Ishaka-Bushenyi, Uganda; 3 Faculty of Biomedical Sciences, Kampala International University, western Campus, Ishaka-Bushenyi, Uganda; 4 Faculty of Health Sciences, Busitema University, Mbale, Uganda

**Keywords:** COVID-19, vaccine, Acceptance, Clinical trials, Western Uganda

## Abstract

**Background:**

Health experts agree that widespread use of safe and effective vaccines will rapidly contain the COVID-19 pandemic. The big question is whether these vaccines can easily be accepted by their end-users. Our study aimed at determining sociodemographic factors associated with acceptance of vaccines and clinical trials of COVID-19 in western Uganda.

**Method:**

A simplified snowball sampling technique was used to select 1067 respondents of 18–70 years in western Uganda using an online questionnaire from July to September 2020. Vaccine acceptability and risk perception were assessed using odds ratio at 95% confidence interval in R software version 3.6.3.

**Results:**

There were 1067 participants in the study. The majority were males (73.2%) and age group 31–40 years (32.6%). The acceptance rate for COVID-19 vaccination was (53.6%; 572/1067) with those aged 18–20 years, males, elites at tertiary level of education (degree or diploma), students, Muslims, married, non-salary earners and rural dwellers having better odds and likeliness to accept vaccination.

Only 44.6% (476/1067) showed interest in clinical trials among which; males, primary school leavers, students, Christians, un-married, respondents who didn’t earn any salary and rural dwellers had better odds and likelihood to participate in clinical trials.

**Conclusion:**

There was a low level of vaccine acceptance and clinical trial interest in western Uganda. Minority groups in the study i.e., Muslims, students, primary school leavers, un-married rural dwellers among others showed more interest in vaccination and clinical trials. We anticipated fears in the larger part of this community that health experts need to address through reassurance of the community that vaccines are tested and that they are safe and important if we are to rapidly contain the COVID-19 pandemic.

**Supplementary Information:**

The online version contains supplementary material available at 10.1186/s12889-021-11197-7.

## Background

Many developed countries have drawn their attention to developing their own vaccine against SARS-CoV-2, the causative agent of COVID-19 [1]. This comes as experts agree that widespread use of safe and effective vaccines will rapidly contain the COVID-19 pandemic thus preventing its transmission [2]. Vaccination programs have been rolled out in many European and Asian countries however Africa is likely to be left behind due to financial constraints as most of these developed countries are now driving up vaccine prices [2].

Currently, the WHO reports that COVID-19 deaths in Africa have surged by 40% ever since the virus was reported on the continent on 14 February 2020. This surge comes as Africa is battling new and more contagious variants for which it has geared up its largest-ever vaccination drive [3]. There are several clinical trials in different parts of the world [4] but according to the African Academy of Sciences, only 2% of these clinical trials take place in African [5]. So far, Egypt and South Africa are the two only African countries currently known to be participating in these trials [6]. According to The Uganda Virus Research Institute [7], Uganda will start its first COVID-19 vaccine trial by December 2021. The institute is reportedly working on a protocol being developed in the United Kingdom by Imperial College London which will focus on vaccine safety and immunogenicity. It is relevant to know that genetic diversity among different populations could affect vaccine efficacy therefore clinical trials within each region or country could be significant.

Much as vaccines are essential in battling against COVID-19, it is paramount to establish vaccine acceptance campaigns before they reach the community. This is because the fear for vaccines has grown radically in the past years [8]. In some African communities, this fear has led to a significant increase in rates of vaccine refusal which has led to an increase in vaccine-preventable diseases [9]. Presently, mistrust in health experts, social propaganda, and myths that most Africans could be immune to the virus are misleading some of the young population in Africa and some of them do not even want to get involved in community health programs now. A recent study among adolescents in Kampala showed adequate knowledge of COVID-19 preventive measures however the uptake of these preventive measures was still low [10]. We should therefore not also forget lessons learned from HIV and Ebola epidemics, that the involvement of communities helped to transform the weak adoption of public health measures [11]. Uganda launched its mass COVID-19 vaccination program on 10th/03/2021 thereby joining a host of countries in Africa to initiate jab inoculations. According to the Ministry of Health, Uganda aims at vaccinating at least 49.6% of its population (21,936,011) with Oxford University–AstraZeneca COVID-19 vaccine at different phases [12]. There has been no mass education about this activity and so many people could be living in fear especially after reading about how similar vaccines have been stopped in some countries all over the world. A recent study in Uganda has shown a low trust of COVID-19 vaccines among healthcare workers, especially those with higher wealth and educational status [13]. The WHO and health experts recommend educational campaigns and endorsement of community leaders as well as healthcare professionals to enhance the likelihood of rapid vaccine uptake [14, 15]. Because of misinformation circulating in the social media creating a lot of mistrust and suspicion around COVID-19 vaccines and clinical trials on the continent [16], our rationale was to identify potential groups most affected. We determined the sociodemographic factors associated with acceptance of vaccination and clinical trials when available, among the people of western Uganda. Our prospective hypothesis was that there would be a high acceptance rate of the vaccine and clinical trial participation among the people of western Uganda.

## Methods

### Study design

In this cross-sectional study, we used a simplified snowball sampling technique to select 1067 respondents from western Uganda. A pretested questionnaire was used to collect data from July to September 2020 among adults of 18–70 years. We designed a questionnaire (Additional file 1) that was administered online through Google forms. Respondents were requested to pass the invitations through emails or WhatsApp contacts in their area. We chose the online method due to limitations of person-to-person contact as a measure to minimize COVID-19 spread. To ascertain quality, the questionnaire was pretested before the final draft was made. In our inclusion criteria, we involved adults of 18 to 70 years of age who had smartphones, and were capable of reading or using the internet. Elderly participants with some challenges of computer or phone use were allowed to seek help from anyone who could be of their help. Participants were required to have internet on either their smartphone or computer in order to answer the questionnaire. Participants were also required to identify their area of residence because our interest was among inhabitants in western Uganda. A consent message was included on the questionnaire and only those that accepted to consent participated in the study Further details of the sample collection procedure are described by Harapan et al. [17].

### Study variables

This study was based on the assumption that the vaccine would freely be availed and provided by the government of Uganda to its people.

The independent variables were the demographic characteristics that included; age, gender, education status, religion, occupation, marital status, monthly income, employment status, occupation, and residence of the respondents.

The dependent variables were responses from the questionnaire about vaccine acceptance and willingness to participate in clinical trials for COVID-19.

For vaccine acceptance: the questions asked were developed in line with Harapan et al [17, 18] which were modified to suit our study. The questions were; 1.1 Do you know the importance of vaccines? 1.2 If the government of Uganda is to provide a free COVID-19 vaccine, would you accept to be vaccinated? 1.3 Do you want to be educated more about the COVID-19 vaccine?

Vaccine trial acceptance: the questions asked were developed according to Harapan et al [17] and they were modified to suit our study design. The questions were; 2.1 Have you ever participated in any vaccine trial before?” “2.2 When approached, would you accept to participate in COVID-19 vaccine trial?”

To minimize bias, we tried to remove unnecessary open-ended questions. The questions were short and clear. The study was designed not to take more than 10 min per respondent as shown in Additional file 1.

### Sample size calculation

With a population size of 8,874,860 in western Uganda [19] a margin of error of 3%, a confidence interval of 95%, and a response distribution of 50% was assumed. The minimum recommended sample size calculated was 1067 individuals.

### Statistical analysis

Data was cleaned and analyzed in R studio software version 3.6.3. Descriptive statistics (frequencies, and percentage) were calculated for the sample demographic characteristics. Contingency tables were drawn and all responses concerning the acceptance of the COVID-19 vaccine; acceptance to participate in vaccine trials were compared against demographic characteristics. Odds Ratios were calculated at 95% confidence interval with data regarded statistically significant only if *P* ≤ 0.05. This was done using Fishers exact two-tailed test.

## Results

### Demographic characteristics of the respondents

There were 1067 participants in the study. The majority were 31–40 years of age (32.6%, 348/1067), males (73.2%, 781/1067), and of the tertiary level of education (degree or diploma) (86.9%, 927/1067). Most of the respondents were also civil servants (39.1%, 417/1067), Christians (88.5%, 944/1067), married (634/1067; 59.4%) who earned a salary of more than 2,000,000 Ugandan shillings ($548) (35.6%, 380/1067). The majority of the respondents also lived in urban centers (65.1%, 695/1067) as shown in Table 1.

**Table 1:** Demographics in the study population

Parameter	Variable	Frequency(***n*** = 1067)	Percentage (%)	95% CI
Age/years	18–20	90	8.4	
	21–30	290	27.2	0.08–0.24
	31–40	348	32.6	0.15–0.47
	41–50	167	15.7	0.17–0.57
	51–60	122	11.4	0.34–1.27
	61–70	50	4.7	0.08–0.36
Sex	Female	286	26.8	
	Male	781	73.2	1.56–2.71
Education	Primary	30	2.8	
	Secondary	110	10.3	1.18–6.66
	Tertiary	927	86.9	1.25–6.11
Occupation	Business	122	11.4	
	Civil servant	417	39.1	0.97–2.17
	Private sector	302	28.3	0.66–1.53
	Retired	13	1.2	0.09–1.34
	Student	193	18.1	1.98–5.15
	Unemployed	20	1.9	0.06–0.75
Religion	Christian	944	88.5	
	Muslim	101	9.5	0.69–1.59
	Others	22	2.1	0.09–0.68
Marital status	Married	634	59.4	
	Not married	433	40.6	0.57–0.93
Monthly Income/SHS	$274–$548	249	23.3	
	<$274	265	24.8	1.10–2.22
	>$548	380	35.6	0.77–1.47
	No salary	173	16.2	1.53–3.44
Residence	Rural	372	34.9	
	Urban	695	65.1	0.61–1.01

### Sociodemographic factors associated with vaccine acceptability intensions

Results show that the acceptance rate for vaccine acceptance intension againstCOVID-19 was (53.6%; 572/1067) (Table 2). Participants in the reference age group 18–20 were more likely to accept the vaccine (OR: 1; 95%CI: NA). Male subjects were twice as likely to accept the vaccine (OR: 2.1; 95%CI: 1.56–2.71; *P* = 0.000). Those who ended at the tertiary level of education and students were more likely to accept the vaccine (OR: 2.8; 95%CI: 1.25–6.11; *P* = 0.009) and (OR: 3.19; 95%CI: 1.98–5.15; *P* = 0.000) respectively. Muslims and non-salary earners were more likely to accept the vaccine (OR: 1.05; 95%CI: 0.69–1.59; *P* = 0.834); (OR: 2.29; 95%CI: 1.53–3.44; *P* = 0.000) respectively.

**Table 2:** Factor associated with vaccine acceptance

Parameters	Variables	Accept vaccine	Percent %	Don’t accept vaccine	Percent %	OR	95% CI	***P***-VALUE
Age/years	18–20	17	18.9	73	81.1	1		
	21–30	182	62.8	108	37.2	0.14	0.08–0.24	0.000
	31–40	163	46.8	185	53.2	0.27	0.15–0.47	0.000
	41–50	72	43.1	95	56.9	0.31	0.17–0.57	0.000
	51–60	32	26.2	90	73.8	0.65	0.34–1.27	0.249
	61–70	29	58.0	21	42.0	0.17	0.08–0.36	0.000
Sex	Female	170	59.4	116	40.6	1		
	Male	325	41.6	456	58.4	2.1	1.56–2.71	0.000
Education	Primary	21	70.0	9	30.0	1		
	Secondary	50	45.5	60	54.5	2.8	1.18–6.66	0.022
	Tertiary	424	45.7	503	54.3	2.8	1.25–6.11	0.009
Occupation	Business	66	54.1	56	45.9	1		
	Civil servant	187	44.8	230	55.2	1.45	0.97–2.17	0.079
	Private sector	163	54.0	139	46.0	1.00	0.66–1.53	1.000
	Retired	10	76.9	3	23.1	0.35	0.09–1.34	0.146
	Student	52	26.9	141	73.1	3.19	1.98–5.15	0.000
	Unemployed	17	85.0	3	15.0	0.21	0.06–0.75	0.012
Religion	Christian	433	45.9	511	54.1	1		
	Muslim	45	44.6	56	55.4	1.05	0.69–1.59	0.834
	Pagans	17	77.3	5	22.7	0.24	0.09–0.68	0.004
Marital status	Married	274	43.2	360	56.8	1		
	Not married	221	51.0	212	49.0	0.73	0.57–0.93	0.012
Monthly Income/SHS	$274–$548	132	53.0	117	47.0	1		
	<$274	111	41.9	154	58.1	1.56	1.10–2.22	0.013
	>$548	195	51.3	185	48.7	1.07	0.77–1.47	0.684
	No salary	57	32.9	116	67.1	2.29	1.53–3.44	0.000
Residence	Rural	158	42.5	214	57.5	1		
	Urban	337	48.5	358	51.5	0.78	0.61–1.01	0.062

Results also showed that (46.4%; 495/1067) were unlikely to accept the vaccine (Table 1). Those aged 61–70 years were unlikely to accept the vaccine (OR: 0.17; 95%CI: 0.08–0.36; *P* = 0.000). Participants who were unemployed and were pagans showed less interest in accepting the vaccine (OR: 0.21; 95%CI: 0.06–0.75; *P* = 0.012) and (OR: 0.24; 95%CI: 0.09–0.68; *P* = 0.004) respectively. The unmarried group and urban dwellers were also unlikely to accept the vaccine (OR: 0.73; 95%CI: 0.57–0.93; *P* = 0.012), and (OR: 0.78; 95%CI: 0.61–1.01; *P* = 0.062) respectively.

### Sociodemographic factors associated with intentions towards vaccine acceptance and clinical trials participation

The acceptance level for participation in COVID-19 clinical trials was (44.6%; 476/1067) (Table 3). Participants in the reference age group 18–20 were more likely to accept clinical trials (OR: 1; 95%CI: NA). Male subjects were 1.1 times likely to accept clinical trials as compared to females (OR: 1.1; 95%CI: 0.84–1.46; *P* = 0.445). Students and civil servants were also more likely to participate in clinical trials compared to the business group (OR: 2.37; 95%CI: 1.49–3.77; *P* = 0.000) and (OR: 1.19; 95%CI: 0.79–1.80; *P* = 0.407) respectively. Participants who were not married and those who had no salary were also more likely to accept participation in clinical trials (OR: 1.3; 95%CI: 1.03–1.69; *P* = 0.028) and (OR: 1.56; 95%CI: 1.05–2.30; *P* = 0.029) respectively. It was also shown that (55.4%; 591/1067) of the respondents were not likely to accept participation in the COVID-19 vaccine clinical trial (Table 3). Participants in the reference age group 21–30 and 61–70 were less likely to accept clinical trials (OR: 0.47; 95%CI: 0.29–0.75; *P* = 0.002) and (OR: 0.18; 95%CI: 0.08–0.41; *P* = 0.000) respectively. Those in the retirement group and the unemployed were also unlikely to participate in the clinical trials (OR: 0.46; 95%CI: 0.12–1.77; *P* = 0.369) and (OR: 0.83; 95%CI: 0.31–2.23; *P* = 0.808) respectively. Muslims and pagans were more unlikely to participate in the clinical trials (OR: 0.59; 95%CI: 0.39–0.92; *P* = 0.020) and (OR: 0.82; 95%CI: 0.34–1.92; *P* = 0.672) respectively. Participants who lived in urban places were also unlikely to participate in clinical trials (OR: 0.69; 95%CI: 0.53–0.89; *P* = 0.004).

**Table 3:** Sociodemographic factors associated with clinical trial acceptance

Parameters	Variables	Accept Clinical trial	Percent %	Don’t accept Clinical trial	Percent %	OR	95% CI	*P*-VALUE
Age/years	18–20	52	57.8	38	42.2	1		
	21–30	113	39.0	177	61.0	0.47	0.29–0.75	0.002
	31–40	167	48.0	181	52.0	0.67	0.42–1.08	0.123
	41–50	83	49.7	84	50.3	0.72	0.43–1.21	0.239
	51–60	51	41.8	71	58.2	0.52	0.30–0.91	0.026
	61–70	10	20.0	40	80.0	0.18	0.08–0.41	0.000
Sex	Female	122	42.7	164	57.3	1		
	Male	354	45.3	427	54.7	1.1	0.84–1.46	0.445
Education	Primary	15	50.0	15	50.0	1		
	Secondary	49	44.5	61	55.5	0.80	0.36–1.80	0.681
	Tertiary	412	44.4	515	55.6	0.80	0.39–1.65	0.552
Occupation	Business	48	39.3	74	60.7	1		
	Civil servant	182	43.6	235	56.4	1.19	0.79–1.80	0.407
	Private sector	119	39.4	183	60.6	1.00	0.65–1.54	1.000
	Retired	3	23.1	10	76.9	0.46	0.12–1.77	0.369
	Student	117	60.6	76	39.4	2.37	1.49–3.77	0.000
	Unemployed	7	35.0	13	65.0	0.83	0.31–2.23	0.808
Religion	Christian	433	45.9	511	54.1	1		
	Muslim	34	33.7	67	66.3	0.59	0.39–0.92	0.020
	Pagans	9	40.9	13	59.1	0.82	0.34–1.92	0.672
Marital status	Married	265	41.8	369	58.2	1		
	Not married	211	48.7	222	51.3	1.3	1.03–1.69	0.028
Monthly Income	$274–$548	115	46.2	134	53.8	1		
	<$274	112	42.3	153	57.7	0.85	0.60–1.21	0.376
	>$548	150	39.5	230	60.5	0.75	0.55–1.04	0.099
	No salary	99	57.2	74	42.8	1.56	1.05–2.30	0.029
Residence/SHS	Rural	188	50.5	184	49.5	1		
	Urban	288	41.4	407	58.6	0.69	0.53–0.89	0.004

## Discussion

This study showed that the level of likeliness to accept the COVID-19 vaccine and willingness to participate in clinical trials among the people of western Uganda islowf4d. This implies that there is mistrust among most community members regarding COVID-19 vaccines and clinical trials which needs to be addressed. Our findings are in agreement with a recent publication among health workers in Uganda [13]. A recent survey on confidence about being vaccinated against COVID-19 in 15 African countries also indicated that work on communication around vaccines is needed [16].

According to WHO, the first COVID-19 vaccination campaigns already started in Ghana and Côte d’Ivoire [20] however many African countries are yet to start this exercise.

Many rumors have previously challenged the success and effectiveness of vaccination programs in Africa. To avoid anti-vaccination campaigns like in the early 1996 and 1997, where the oral polio vaccine was criticized by the public for being contaminated with HIV and ineffective, the Ugandan government needs proper mobilization and community engagement in this vaccination exercise [21, 22]. In 2019, the Congo’s Ebola vaccine hesitancy was geared by community mistrust due to social and cultural factors that arose during the West Africa Ebola outbreak, even though these communities seriously needed the Ebola vaccine [23]. In the United States of America, vaccine reluctance led to a measles outbreak among the vulnerable unimmunized children in urban centers [24]. Vaccine refusal is associated with increases in illness and death from vaccine-preventable diseases which is also secondary to large economic costs for health care [9].

From this study, we observed that there were several factors associated with vaccine acceptance.

Young respondents below 20 years for instance were more willing to be vaccinated than any other age group but this age group was one of the least participating groups in this study. This, points out that there could be fear arising among other age groups regarding vaccination. Male respondents also showed more interest in receiving the vaccine than the females. It is practically known in Uganda that ladies are often more cooperative in health activities than men but this was different in our findings. This was also a possible indicator of fear for the vaccination among the female gender. In addition, we believed that social media could have played a role in giving false confidence that the African continent is immune to COVID-19 because of its climatic conditions. Some people could have lost interest in vaccination basing on this argument [25] while others could be worried about vaccine side effects [16].

The elites at tertiary level of education were more enthusiastic about receiving the vaccine. This particular group is taken to be knowledgeable in society. Their willingness was unquestionable since they are assumed to know the outcomes of not being vaccinated. Other studies have shown that low-level education is a valid indicator for vaccine refusal [26].

There were more Christians (88.5%) than Muslims (9.5%) among the respondents however Muslims showed more interest in vaccine acceptance. Most of the churchgoers were hesitant about vaccination. In some countries like Nigeria, some Christians have referred to the COVID-19 vaccine as being a mark of the beast [27]. Previously in northern Nigeria, religious leaders had a wrong perception about the polio vaccination program [28]. Their rejection of vaccination programs exposed more individuals to infectious illnesses and this led to disease progression within the community. The government, therefore, needs to involve religious leaders in community sensitization about the COVID-19 vaccine.

We also observed that there was a low rate of likeliness to participate in vaccine clinical trials. It should be noted that the success or failure of any vaccine trial is community-driven [29]. Among those willing to participate in COVID-19 clinical trials were youth of ages 18–20 years, males, students, civil servants, and business personnel. Young adults of < 20 years were among the least participants in the study. We believed that majority of the respondents because they had never participated in clinical trials had fear for adverse side effects of the drug to be tested. Lack of sensitization about the need to get involved in vaccine clinical trials could have affected the likelihood to participate in vaccine trials.

Previous research in Uganda showed that before the start of any trial, researchers should first gain support from politicians, the media and the general public [30]. Now that the UVRI, Uganda is set to roll out its first COVID-19 clinical trials in December 2021, the government of Uganda needs to know that community mobilization will heavily contribute to increased uptake of the prospective COVID-19 clinical trials when available. The reason is that community participation promotes a sense of ownership of any health activity [25]. We, suggest that to improve participation, health experts should ensure that required information is comprehensively explained to the participants and that the important aspects of study participation are emphasized.

The government can establish messages and training for its people through radios, televisions, newspapers, seminars, and phone messages.

## Conclusion

The level of likeliness to accept COVID-19 vaccination and willingness to participate in clinical trials was low in western Uganda. The minority group i.e.; the young [25, 26, 28], Muslims, students, primary school leavers, non-salaried persons, un-married, and rural dwellers were more interested in vaccination and clinical trials. We anticipated fears among the community that health experts need to address through reassurance of the community that vaccines are tested and that they are safe and important if we are to rapidly contain the COVID-19 pandemic. More researches should be done in different parts of the country to ascertain the readiness of the community for vaccination.

### Study limitations

With this study being purely an online-based survey, it was not possible to know if the responses were genuine. The study being online-based could also have omitted those without phones, computers, and the internet. We could have left out more vulnerable persons or persons who could have preferred vaccination hence the low level of acceptance obtained.

## Supplementary Information

**Additional file 1.** Study questionnaire.

## Data Availability

Data used is accessible on fig share through the link: https://figshare.com/s/46d3ef8e7c4553f3c4e6

## Abbreviations

%: Percentage
CI: Confidence interval
COVID-19: Coronavirus disease 2019
HIV: Human Immune Virus
OR: Odds ratio
*P-*Value: Probability value
UVRI: Uganda Virus Research Institute
WHO: World Health Organization
