# Supplementary material for: Sociodemographic factors associated with acceptance of COVID-19 vaccine and clinical trials in Uganda: a cross-sectional study in western Uganda
Source: BMC Public Health. 2021 Jun 10;21:1106. doi: 10.1186/s12889-021-11197-7 (PMC8190743; doi:10.1186/s12889-021-11197-7)
Supplement: Supplementary file 1 — Additional file 1. Study questionnaire. [file 12889_2021_11197_MOESM1_ESM.docx]

**Additional file 1:** **Study questionnaire**

**Introduction statement**

According to the East African Consortium for Clinical Research, the Uganda Virus Research Institute (UVRI) is partnering with Imperial College London to start the first Covid-19 vaccine trial in the country. However, the success or failure of any vaccine program is community driven. The aim of this study is to determine sociodemographic factors associated with acceptance of vaccines and clinical trials of COVID-19 in western Uganda

Principle Investigator: Echoru Isaac, Kabale University

Tel: +256788089078

E-mail: [iechoru@kab.ac.ug](mailto:iechoru@kab.ac.ug)

**Consent**

I voluntarily agree to participate in this research study. I understand that even if I agree to participate now, I can withdraw at any time or refuse to answer any question without any consequences of any kind. I have read the purpose of this research. I understand that in any report on results of this research my identity will remain anonymous.

Filling this questionnaire means you have given consent.

**SECTION A: Demographic data**

1. Identify your Age group?
2. 18-20
3. 21-30
4. 31-40
5. 41-50
6. 51-60
7. 61-70
8. What is your Gender?
9. Male
10. Female
11. What is your education level?
12. Primary
13. Secondary
14. Tertiary (institutional college or university)
15. What your occupation category?
16. Business person
17. Civil servant (government employee)
18. Private sector
19. Retired person
20. Still a Student
21. Unemployed
22. What is your religious background?
23. Christian (catholic, protestant, born again, orthodox, Adventist, Jehovah’s Witness)
24. Muslim
25. Pagan (I don’t believe in any religion or traditional believer)
26. What is your marital status?
27. Married
28. Unmarried (single or divorced)
29. What is your monthly income?
30. 1,000,000-2,000,000
31. Less than 1,000,000
32. More than 2,000,000
33. No monthly salary
34. What is the nature of your residence?
35. Rural
36. Urban

**SECTION B:** The following questions are about vaccine acceptance, acceptance to participate in clinical trials and hypothetical risk perception of COVID-19 vaccine. You are required to choose either YES or NO option.

1. Do you know the importance of vaccines?
   1. YES
   2. NO
2. Vaccine acceptance: “If the government of Uganda is to provide free COVID-19 vaccine, would you accept to be vaccinated?”
3. YES
4. NO
5. Do you need more education about vaccines?
   1. YES
   2. NO
6. Vaccine trial acceptance: “Have you ever participated in any vaccine trial before?”
7. YES
8. NO
9. “When approached, would you accept to participate in COVID-19 vaccine trial?”
10. YES
11. NO
12. Do you know the risks of not being vaccinated?
13. YES
14. NO
15. Risk perception of the vaccine: “Even before COVID-19 vaccine is available, what is your risk perception about this vaccine?”
16. HIGH
17. MODERATE
18. LOW
